# Post-transplant malignancies in Taiwanese kidney transplant recipients: incidence, temporal trends, and immunosuppressive risk factors in a two-decade single-center cohort

**DOI:** 10.3389/fimmu.2026.1738516

**Published:** 2026-08-17

**Authors:** Hsiang-Chen Hsieh, Jian-Ri Li, Shian Shiang Wang, Chuan-Shu Chen, Chun-Kuang Yang, Shun-Fa Yang

**Affiliations:** 1 Institute of Medicine, Chung Shan Medical University, Taichung, Taiwan; 2 Department of Urology, Taichung Veterans General Hospital, Taichung, Taiwan; 3 Department of Post-Baccalaureate Medicine, College of Medicine, National Chung Hsing University, Taichung, Taiwan; 4 Department of Medicine and Nursing, Hungkuang University, Taichung, Taiwan; 5 Department of Applied Chemistry, National Chi Nan University, Nantou, Taiwan; 6 Department of Biological Science and Technology, National Yang Ming Chiao Tung University, Hsinchu, Taiwan; 7 Department of Medical Research, Chung Shan Medical University Hospital, Taichung, Taiwan

**Keywords:** immune surveillance, immunosenescence, immunosuppression, kidney transplantation, post-transplant malignancy, tacrolimus, Taiwan, urothelial carcinoma

## Abstract

**Background:**

Post-transplant malignancies (PTMs) remain major late immune-related complications after kidney transplantation, but long-term Asian data integrating immunologic exposures are limited.

**Methods:**

We retrospectively analyzed 1,808 kidney transplant recipients from 2002 to 2022 at Taichung Veterans General Hospital, Taiwan. After exclusions, 1,734 patients were eligible. Outcomes included PTM incidence, spectrum, latency, temporal trends, and survival, with prespecified analyses of longitudinal tacrolimus trough levels and cumulative immunosuppressive burden.

**Results:**

PTM incidence was 11.7%. Urothelial (bladder 15.8%, upper tract 14.3%) and liver cancers predominated. One third occurred within 5 years with a median latency of 8.2 years. Bladder cancer declined after 2012 (26.7% *vs*. 11.2%, *p* = 0.010). Older age independently predicted PTM (HR 1.08 per year, *p* < 0.001) and mortality (HR 1.07 per year, *p* < 0.001). Higher long-term tacrolimus trough levels were independently associated with mortality in both the overall and PTM cohorts (HR 1.24 and 1.37, both p < 0.05). PTM patients had higher mortality (46.3% *vs*. 30.6%) with median post-diagnosis survival of 16.5 months.

**Conclusions:**

PTMs in Taiwan are frequent and immunologically shaped, with a distinct urothelial and hepatic predominance and a post 2012 decline in bladder cancer plausibly linked to evolving immunosuppression, surveillance, and reduced carcinogen exposure. Older age and cumulative immunosuppressive burden were associated with PTM development, whereas higher long-term tacrolimus trough levels independently predicted mortality, underscoring the need for individualized immunosuppressive management and cancer type specific surveillance.

## Introduction

Kidney transplantation is widely regarded as the gold standard treatment for patients with end-stage kidney disease (ESKD), significantly improving survival and quality of life compared with dialysis (1–3). However, post transplant malignancies (PTMs) remain one of the most serious long term complications, ranking just behind cardiovascular disease and infection in most registries (4–7). The overall cancer risk among kidney transplant recipients (KTRs) is estimated to be two to threefold higher than in the general population (4, 5, 7–10), reflecting the combined effects of chronic immunosuppression, impaired immune surveillance, and oncogenic viral reactivation (5, 9–11).

The spectrum of PTMs differs markedly by geography. In Western countries, non melanoma skin cancers and lymphomas predominate (5, 12–17), whereas urothelial, gastric, hepatic, and renal cancers are more prevalent in Asian populations (3, 18–23). In Taiwan, previous studies have highlighted urothelial and liver cancers as particularly common PTM subtypes (18, 24–26). These discrepancies likely reflect a combination of genetic predispositions and environmental exposures unique to Asia, including aristolochic acid containing herbal remedies (21, 22), arsenic contaminated groundwater (23), and high endemic rates of chronic hepatitis B and C infection (2, 7, 11).

Immunosuppressive therapy is a central driver of oncogenesis after transplantation. Calcineurin inhibitors, antimetabolites, and corticosteroids each impair tumor immune surveillance through suppression of cytotoxic CD8^+^ T cell and NK cell activity, altered cytokine signaling, and reduced antigen presentation (7–10, 31–33). Antibody therapies further increase the risk of post transplant lymphoproliferative disorder, whereas mTOR inhibitors may confer partial protection through antiproliferative and immune modulatory effects (2, 8, 18, 19, 35). Increasing evidence suggests that the cumulative immunosuppressive burden rather than any single agent is the dominant determinant of malignancy risk (7, 10, 36–38). Tacrolimus, the most widely prescribed calcineurin inhibitor, has been associated with dose- and context-dependent oncogenic effects through TGF-β and mTOR signaling pathways (32–34), although cumulative immunosuppressive exposure rather than tacrolimus concentration alone appears to be the more important determinant of long-term malignancy risk (10, 36). Recent mechanistic work indicates that sustained calcineurin inhibition reprograms effector and antigen presenting cell metabolism, promoting immune dysfunction that weakens antitumor surveillance (20).

Despite the clinical importance of PTMs, long term Asian data integrating immunologic and epidemiologic factors remain limited. Registry studies from Korea, Hong Kong, Iran, and Japan have consistently reported lower incidences than Western cohorts, but with shorter observation windows and minimal evaluation of longitudinal immunosuppressive exposure (14–17, 24–26). Nationwide analyses in Taiwan from 1997 to 2008 estimated PTM incidences between 6.8% and 8.4% (10, 24–26). However, these earlier datasets predated modern immunosuppressive protocols and major environmental interventions, leaving the long term interplay between immune modulation, carcinogen exposure, and cancer risk poorly defined. Contemporary syntheses emphasize that robust, long horizon Asian datasets are needed to clarify how evolving immunosuppressive strategies and regional exposures shape cancer risk in transplant recipients (6).

The present study leverages a two-decade, single center cohort of kidney transplant recipients in Taiwan to address these gaps. We aimed to characterize the incidence and spectrum of PTMs, analyze temporal trends in cancer epidemiology, and identify key demographic and immunosuppressive risk factors associated with malignancy and mortality. Through this analysis, we sought to improve understanding of long-term PTM epidemiology and immunosuppressive exposure in kidney transplant recipients, thereby informing individualized immunosuppressive management and cancer type-specific surveillance strategies.

## Materials and methods

### Study design and population

We conducted a retrospective cohort study based on the medical records of all adult patients who underwent kidney transplantation at Taichung Veterans General Hospital, a tertiary referral center in central Taiwan, between January 2002 and January 2022. A total of 1,808 recipients were screened. After excluding those with pre-transplant malignancy, cancer diagnosed within 3 months post-transplant, recurrent or multiple malignancies, incomplete records, or follow-up less than 12 months, 1,734 patients were included in the analytic cohort. Of these, 203 recipients developed post-transplant malignancy (PTM).

### Ethics approval

The study protocol was approved by the Institutional Review Board of Taichung Veterans General Hospital (IRB No. CE22425A-2). As this was a retrospective study using de-identified clinical data, the requirement for written informed consent was waived by the Institutional Review Board in accordance with institutional policy and local regulations. All data were anonymized prior to analysis in accordance with institutional and national data protection regulations. All study procedures were conducted in accordance with the principles of the Declaration of Helsinki.

### Data collection and definitions

Demographic and clinical variables included age at transplantation, sex, comorbidities, donor type, and follow-up duration. Malignancy diagnoses were confirmed by histopathology and classified according to ICD codes and histological subtypes. Mortality data were validated using hospital records and linkage with the national death registry.

Immunosuppressive regimens were reviewed in detail, and ever-use of common agents (tacrolimus, cyclosporine, sirolimus, everolimus, mycophenolate mofetil, methylprednisolone, prednisolone, and hydrocortisone) was documented.

Analyses of immunosuppressive regimens were performed in recipients with sufficient medication records to characterize immunosuppressive drug combinations (n = 1,476). Longitudinal tacrolimus exposure analyses were conducted separately in recipients with complete serial FK506 trough measurements (n = 625).

### Tacrolimus exposure assessment

Tacrolimus trough levels were routinely measured as part of standard post-transplant therapeutic drug monitoring. The index date was defined as the date of PTM diagnosis for recipients who developed PTM and the last available follow-up date for recipients without PTM. Long-term tacrolimus exposure was assessed using all available through concentrations measured from kidney transplantation to the index date, whereas short-term exposure was summarized using trough concentrations measured during the 90 days preceding the index date. For descriptive analyses, long-term (LFK) and short-term (SFK) tacrolimus exposure were first summarized for each recipient as the mean tacrolimus trough concentration over the corresponding observation period. These patient-level values were subsequently summarized as the median (interquartile range) for each study group. For regression analyses, four summary metrics (mean, median, minimum, and maximum trough concentrations) were evaluated separately as candidate exposure variables (designated LFK1–LFK4 and SFK1–SFK4, respectively). Tacrolimus exposure was analyzed as a continuous variable; no predefined therapeutic threshold, cumulative area-under-the-curve (AUC), or time-weighted exposure metric was used.

### Prespecified subgroup analyses

Two prespecified subgroup analyses were performed. Longitudinal tacrolimus exposure was evaluated in 625 recipients with complete serial FK506 trough data available throughout the observation period, among whom 62 developed PTM.

A paired pre- and post-PTM tacrolimus analysis was subsequently performed in 44 PTM recipients with at least one year of FK506 trough data available both before and after PTM diagnosis, allowing paired comparison of median tacrolimus trough levels during the one-year periods before and after PTM diagnosis.

### Outcomes

Primary outcomes were the incidence, spectrum, and latency of PTM. Secondary outcomes included overall survival, cancer-specific survival, and risk factors associated with PTM development and all-cause mortality. Additional analyses evaluated the associations between tacrolimus exposure, cumulative immunosuppressive burden, and these clinical outcomes.

### Statistical analysis

Continuous variables were summarized as mean ± standard deviation or median (interquartile range) and compared using the Mann–Whitney U test. Categorical variables were analyzed with the Chi-square test or Fisher’s exact test, as appropriate. Tacrolimus levels before and after PTM were compared using the Wilcoxon signed-rank test. Cox proportional hazards regression was used to identify independent predictors of PTM and mortality, with results reported as hazard ratios (HRs) and 95% confidence intervals (CIs). All statistical tests were two-sided, and a p value <0.05 was considered statistically significant.

### Statistical analysis data availability statement

The datasets generated for this study are available from the corresponding author upon reasonable request.

## Results

### Patient cohort

**Figure 1:**
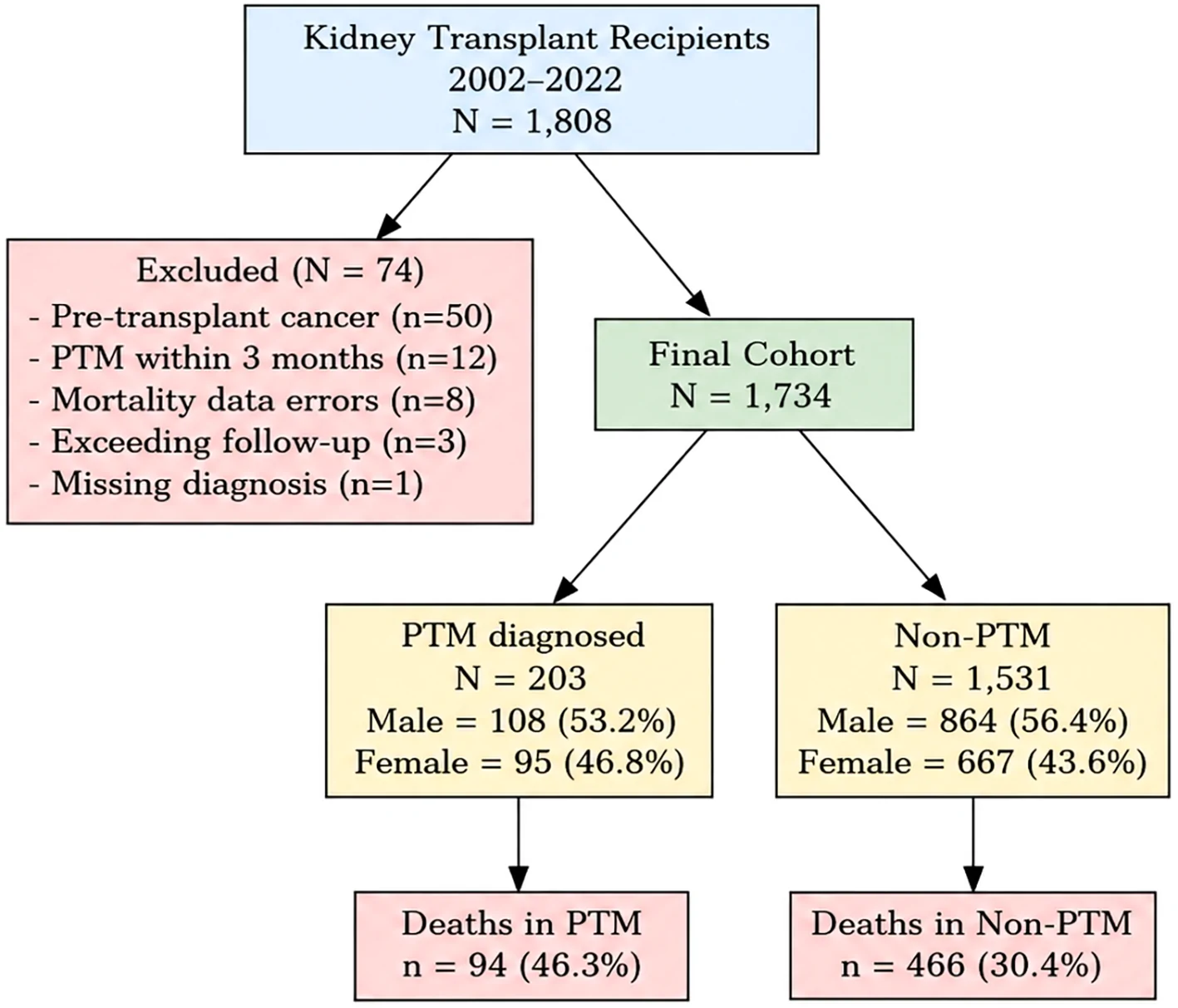
Flowchart of patient inclusion and exclusion in the kidney transplant cohort. A total of 1,808 kidney transplant recipients were screened; 74 were excluded. The final cohort included 1,734 patients, of whom 203 developed PTM and 1,531 did not.

Of 1,808 kidney transplant recipients screened, 74 were excluded (50 with pre-transplant cancer, 12 with early PTM < 3 months, 8 with inconsistent mortality records, 3 exceeding the observation window, and 1 with incomplete data). The final analytic cohort comprised 1,734 patients, of whom 203 (11.7%) developed post-transplant malignancy (PTM) (**Figure 1**). Baseline characteristics are shown in **Table 1**. Patients who developed PTM were significantly older at transplantation (median 52 *vs*. 46 years, p < 0.001), whereas sex distribution was similar. Mortality occurred more frequently in PTM patients than in non-PTM recipients (46.3% *vs*. 30.6%, p < 0.001).

**Table 1:** Baseline characteristics of kidney transplant recipients with and without PTM.

Variable	PTM (N = 203)	Non-PTM (N = 1,531)	Total (N = 1,734)	p-value
age_KT, Mean ± SD	51.0 ± 11.7	46.2 ± 13.0	46.7 ± 13.0	<0.001
age_C, Median(IQR)	60.0 (54.0–67.0)	–	–	–
Sex – Female, n (%)	95 (46.8)	667 (43.6)	762 (43.9)	0.43
Sex – Male, n (%)	108 (53.2)	864 (56.4)	972 (56.1)	
Pre-KT Comorbidities				
DM	31 (15.3)	220 (14.4)	251 (14.5)	0.813
HTN	78 (38.4)	509 (33.2)	587 (33.9)	0.166
HBV	11 (5.4)	46 (3.0)	57 (3.3)	0.109
HCV	6 (3.0)	21 (1.4)	27 (1.6)	0.158
After-KT Comorbidities				
DM	61/172 (35.5)	324/1312 (24.7)	385/1484 (26.0)	0.003
HTN	74/125 (59.2)	458/1023 (44.8)	532/1148 (46.4)	0.003
HBV	32/192 (16.7)	108/1486 (7.6)	140/1678 (8.4)	<0.001
HCV	28/197 (14.2)	72/1511 (5.0)	100/1708 (5.9)	<0.001
Overall Comorbidities				
DM	92 (45.3)	544 (35.5)	636 (36.7)	0.008
HTN	152 (74.9)	967 (63.2)	1,119 (64.5)	0.001
HBV	43 (21.2)	154 (10.1)	197 (11.4)	<0.001
HCV	34 (16.8)	93 (6.1)	127 (7.3)	<0.001
Mortality, n (%)	94 (46.3)	466 (30.4)	560 (32.3)	<0.001
time2, Mean ± SD	13.2 ± 6.1	11.1 ± 6.9	11.4 ± 6.9	<0.001
time1, Median(IQR)	8.2 (3.6–12.3)	–	–	–

Age_KT, age at kidney transplantation; age_C, age at cancer diagnosis; KT, kidney transplantation; PTM, post-transplant malignancy; SD, standard deviation; IQR, interquartile range; HBV, hepatitis B virus; HCV, hepatitis C virus; DM, diabetes mellitus; HTN, hypertension.

Statistical methods: Continuous variables were compared using t-test or Mann–Whitney U test, and categorical variables using Chi-square or Fisher’s exact test, as appropriate. A p-value <0.05 was considered statistically significant.

### Incidence and spectrum of PTM

**Figure 2:**
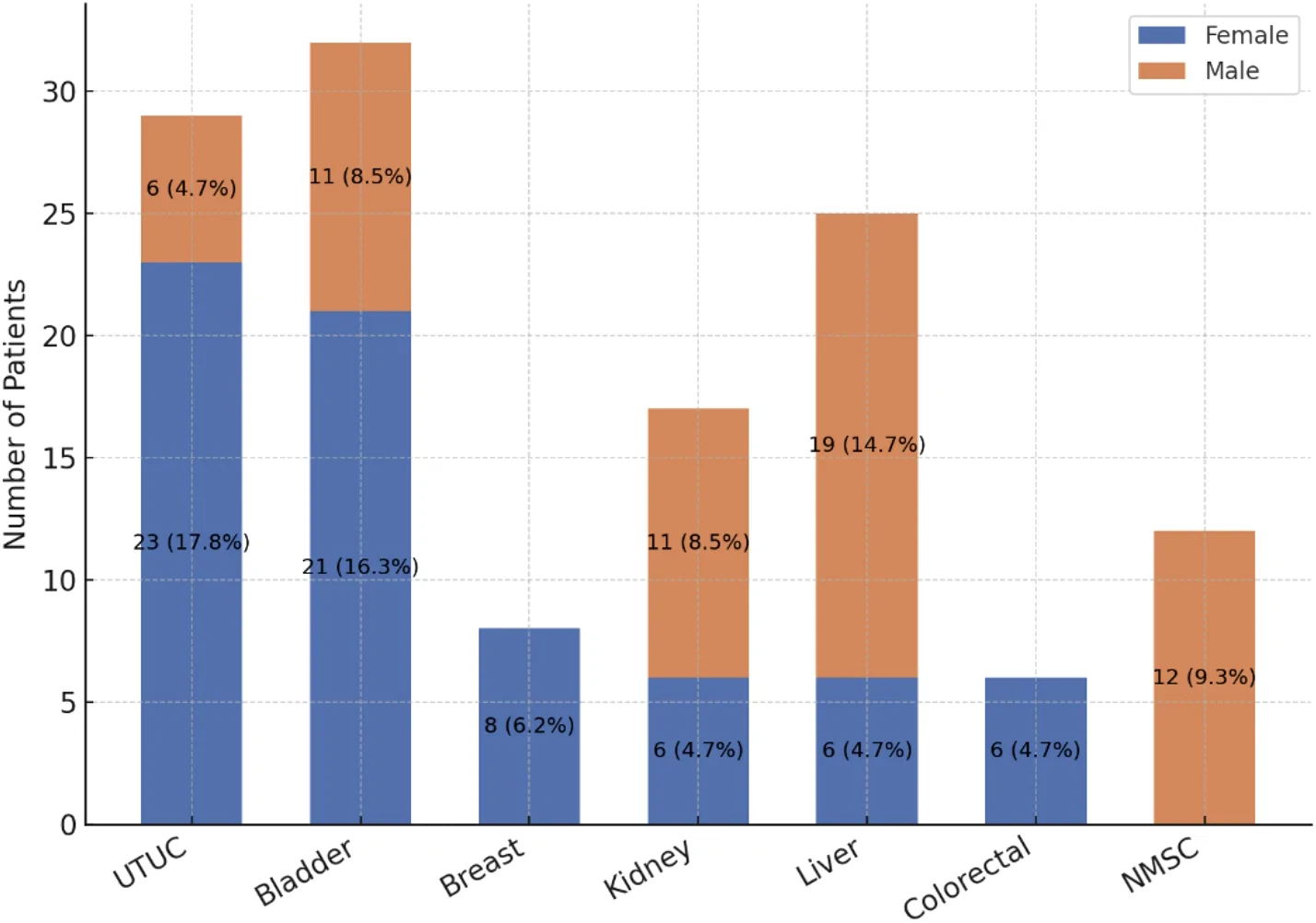
Distribution of major post-transplant malignancy (PTM) subtypes by sex. Stacked bar chart showing sex-specific distribution of urothelial, bladder, breast, kidney, liver, colorectal, and non-melanoma skin cancers. Blue indicates female recipients; orange indicates male recipients.

During a median follow-up of 12.1 years, the overall incidence of PTM was 11.7%. The most common cancers were bladder cancer, upper tract urothelial carcinoma (UTUC), and liver cancer, followed by kidney cancer and non-melanoma skin cancer (**Table 2**). The proportional distribution of PTM subtypes is illustrated in **Supplementary Figure 1**. Urothelial cancers predominated in female recipients, whereas liver cancer and non-melanoma skin cancer (NMSC) were more common in males (**Figure 2**; **Supplementary Figure 2**).

**Table 2:** Distribution of post-transplant malignancy types by sex (N = 203).

Cancer type	Female (N = 95)	Male (N = 108)	Total (N = 203)
Bladder	21 (22.1%)	11 (10.2%)	32 (15.8%)
Liver	6 (6.3%)	19 (17.6%)	25 (12.3%)
Kidney	6 (6.3%)	11 (10.2%)	17 (8.4%)
Skin (NMSC)	5 (5.3%)	12 (11.1%)	17 (8.4%)
Ureter	12 (12.6%)	4 (3.7%)	16 (7.9%)
Renal pelvis	11 (11.6%)	2 (1.9%)	13 (6.4%)
Colorectal	6 (6.3%)	9 (8.3%)	15 (7.4%)
Lung	3 (3.2%)	8 (7.4%)	11 (5.4%)
Breast	8 (8.4%)	0 (0.0%)	8 (3.9%)
Prostate	0 (0.0%)	8 (7.4%)	8 (3.9%)
Thyroid	1 (1.1%)	4 (3.7%)	5 (2.5%)
Brain	3 (3.2%)	1 (0.9%)	4 (2.0%)
Pancreas	0 (0.0%)	4 (3.7%)	4 (2.0%)
Cervix	3 (3.2%)	0 (0.0%)	3 (1.5%)
Vulva	3 (3.2%)	0 (0.0%)	3 (1.5%)
Unknown primary	1 (1.1%)	2 (1.9%)	3 (1.5%)
Endometrium	2 (2.1%)	0 (0.0%)	2 (1.0%)
Stomach	1 (1.1%)	1 (0.9%)	2 (1.0%)
Lymph node	1 (1.1%)	1 (0.9%)	2 (1.0%)
Parotid gland	0 (0.0%)	1 (0.9%)	1 (0.5%)
Penis	0 (0.0%)	1 (0.9%)	1 (0.5%)
Thoracic esophagus	0 (0.0%)	1 (0.9%)	1 (0.5%)
Nasopharynx	1 (1.1%)	0 (0.0%)	1 (0.5%)
Oropharynx	0 (0.0%)	1 (0.9%)	1 (0.5%)
Cheek (malignant neoplasm)	0 (0.0%)	1 (0.9%)	1 (0.5%)
Mouth (malignant neoplasm)	0 (0.0%)	1 (0.9%)	1 (0.5%)
Tongue	0 (0.0%)	1 (0.9%)	1 (0.5%)
Bone marrow	1 (1.1%)	0 (0.0%)	1 (0.5%)
Soft tissue	0 (0.0%)	1 (0.9%)	1 (0.5%)
Extrahepatic bile duct	0 (0.0%)	1 (0.9%)	1 (0.5%)
Gallbladder	0 (0.0%)	1 (0.9%)	1 (0.5%)
Intrahepatic bile duct	0 (0.0%)	1 (0.9%)	1 (0.5%)

PTM, post-transplant malignancy; NMSC, non-melanoma skin cancer. Percentages are calculated within each sex group.

The median latency from transplantation to PTM diagnosis was 8.2 years (IQR 3.8–12.3). Approximately one third of cases occurred within 5 years, including 5.9% within 1 year and 18.7% within 3 years (**Supplementary Figure 3**). Latency varied by subtype: urothelial and hepatic cancers developed earlier, while kidney cancer occurred later (**Supplementary Table 1**). Cumulative incidence curves stratified by cancer type are shown in **Supplementary Figure 4**.

### Temporal trends

**Figure 3:**
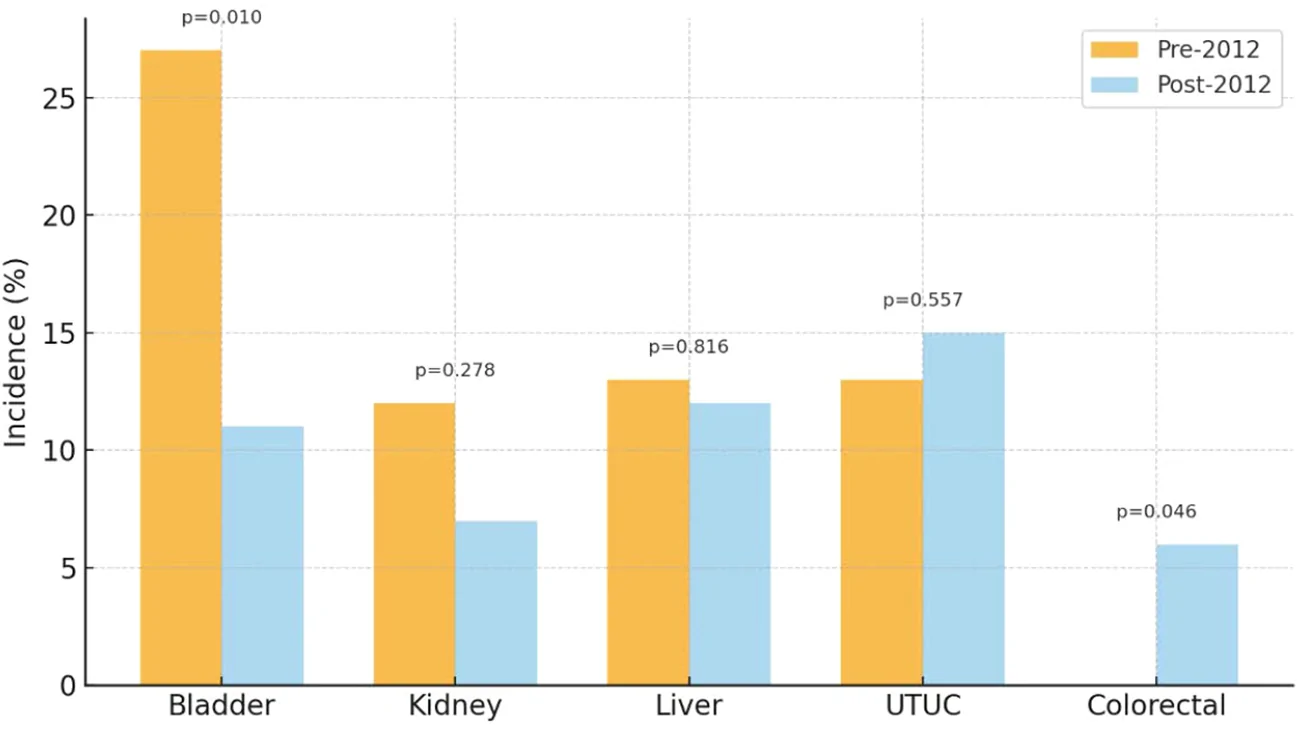
Temporal distribution of major post-transplant malignancy (PTM) subtypes before and after 2012. Bar chart comparing incidences of bladder, kidney, liver, upper tract urothelial carcinoma (UTUC), and colorectal cancers between pre-2012 and post-2012 cohorts. P values for between-group comparisons are shown above each pair of bars.

Between the pre-2012 and post-2012 eras, several notable changes were observed. The incidence of bladder cancer declined significantly after 2012 (26.7% *vs*. 11.2%; OR 0.35, 95% CI 0.16–0.75, p = 0.010), whereas liver, kidney, and skin cancer incidences remained stable. Colorectal cancer appeared only in the post-2012 cohort (6.0% *vs*. 0%, p = 0.060). The median age at cancer diagnosis increased significantly over time (56.5 *vs*. 62.0 years, p < 0.001). These results are summarized in **Supplementary Table 2** and illustrated in **Figure 3**.

### Risk factors for PTM and mortality

In the tacrolimus exposure cohort (n = 625), PTM patients were significantly older at transplantation and had higher mortality than non-PTM recipients (**Table 3A**). Both long-term (LFK) and short-term (SFK) mean tacrolimus trough concentrations were significantly lower in PTM recipients than in non-PTM recipients (**Table 3B**). In the paired subgroup of 44 PTM recipients, median tacrolimus trough levels were significantly lower during the year after PTM diagnosis than during the year before diagnosis (4.6 *vs*. 4.7 ng/mL, p = 0.007). In the immunosuppressive regimen cohort (n = 1,476), PTM recipients were more likely to have received five or more immunosuppressive agents, and greater immunosuppressive burden was associated with an increased risk of PTM. Hydrocortisone and sirolimus were more frequently used among PTM recipients, whereas standard triple therapy with tacrolimus, mycophenolate, and corticosteroids did not differ significantly between groups (**Table 3C**; **Supplementary Table 3**).

**Table 3:** Clinical characteristics and immunosuppressive exposure associated with post-transplant malignancy (PTM).

Part A. Baseline demographics, comorbidities, and follow-up (N = 625)				
Variable	PTM (N = 62)	Non-PTM (N = 563)	Total (N = 625)	p-value
Age_KT, mean ± SD (y)	49.9 ± 11.6	44.5 ± 12.4	45.1 ± 12.4	<0.001
Age_C, median (IQR) (y)	60.0 (54–67)	–	–	–
Mortality, n (%)	15 (24.2)	62 (11.0)	77 (12.3)	0.003
Pre-KT comorbidities, n (%)				ns
DM	10 (16.1)	77 (13.7)	87 (13.9)	0.62
HTN	23 (37.1)	190 (33.7)	213 (34.1)	0.63
After-KT comorbidities, n (%)				ns
DM	12 (19.4)	93 (16.5)	105 (16.8)	0.55
HTN	14 (22.6)	125 (22.2)	139 (22.2)	0.94
Overall comorbidities, n (%)				ns
DM	22 (35.5)	170 (30.2)	192 (30.7)	0.39
HTN	37 (59.7)	315 (55.9)	352 (56.3)	0.58
Time1 (KT→Cancer), median (IQR), y	12.0 (7.5–15.0)	–	–	–
Time2 (Follow-up), mean ± SD, y	14.4 ± 5.9	12.6 ± 6.6	12.8 ± 6.6	0.02
Part B. Tacrolimus exposure				
Variable	PTM	Non-PTM	p-value	
LFK (median [IQR], ng/mL)	5.18 (3.1–6.7)	5.90 (4.9–7.0)	0.016	
SFK (median [IQR], ng/mL)	5.22 (3.4–6.4)	5.74 (4.6–7.2)	0.014	
Subgroup (N = 44) FK506 trough before *vs* after PTM				
1 year before PTM	4.7 (3.6–6.3)	–	–	
1 year after PTM	4.6 (3.0–5.5)	–	0.007	
Part C. Immunosuppressive regimens (N = 1476)				
Number of drugs (overall χ² = 25.05, p < 0.001)				
Number of drugs	PTM (N = 195)	Non-PTM (N = 1281)	Total (N = 1476)	
1 drug	9 (4.62%)	162 (12.65%)	171 (11.59%)	
2 drugs	27 (13.85%)	217 (16.94%)	244 (16.53%)	
3 drugs	48 (24.62%)	318 (24.82%)	366 (24.80%)	
4 drugs	51 (26.15%)	353 (27.56%)	404 (27.37%)	
5 drugs	52 (26.67%)	207 (16.16%)	259 (17.55%)	
6 drugs	8 (4.10%)	24 (1.87%)	32 (2.17%)	
Drug combinations (overall χ² = 74.09, p < 0.001)				
Regimen	PTM (N = 195)	Non-PTM (N = 1281)	Total (N = 1476)	
Hydrocortisone	6 (3.08%)	58 (4.53%)	64 (4.34%)	
Mycophenolate	3 (1.54%)	66 (5.15%)	69 (4.67%)	
Tacrolimus + Mycophenolate	9 (4.62%)	135 (10.54%)	144 (9.76%)	
Hydrocortisone + Tacrolimus + Mycophenolate	19 (9.74%)	47 (3.67%)	66 (4.47%)	
Prednisolone + Tacrolimus + Mycophenolate	5 (2.56%)	141 (11.01%)	146 (9.89%)	
Tacrolimus + Mycophenolate + Sirolimus	14 (7.18%)	55 (4.29%)	69 (4.67%)	
Methylpred + Hydrocortisone + Tacrolimus + Mycophenolate	9 (4.62%)	57 (4.45%)	66 (4.47%)	
Methylpred + Prednisolon + Tacrolimus + Mycophenolate	9 (4.62%)	135 (10.54%)	144 (9.76%)	
Hydrocortisone + Tacrolimus + Mycophenolate + Sirolimus	10 (5.13%)	47 (3.67%)	57 (3.86%)	
Methylpred + Hydrocortisone + Prednisolon + Tacrolimus + Mycophenolate	12 (6.15%)	120 (9.37%)	132 (8.94%)	
Methylpred + Hydrocortisone + Tacrolimus + Mycophenolate + Sirolimus	18 (9.23%)	39 (3.04%)	57 (3.86%)	
Others	81 (41.54%)	381 (29.74%)	462 (31.30%)	

(A) Baseline demographics, comorbidities, and follow-up characteristics of kidney transplant recipients with and without PTM (N = 625).

(B) Tacrolimus exposure, including long-term tacrolimus trough levels (LFK; patient-level mean trough concentration from kidney transplantation to the index date, summarized as the median [IQR] for each study group), short-term tacrolimus trough levels (SFK; patient-level mean trough concentration during the 90 days preceding the index date, summarized as the median [IQR] for each study group), and paired subgroup analysis comparing median tacrolimus trough levels during the 1-year periods before and after PTM diagnosis (N = 44).

(C) Immunosuppressive regimens among recipients with sufficient medication records to permit characterization of immunosuppressive drug combinations (N = 1,476), stratified by the number of immunosuppressive agents and by specific drug combinations. Overall chi-square tests demonstrated significant differences between PTM and non-PTM groups in both number of drugs (χ² = 25.05, p < 0.001) and drug combinations (χ² = 74.09, p < 0.001).

KT, kidney transplantation; PTM, post-transplant malignancy; age_KT, age at kidney transplantation; age_C, age at cancer diagnosis; FK506, tacrolimus; LFK; patient-level mean tacrolimus trough concentration from kidney transplantation to the index date, summarized as the median (IQR) for each study group; SFK; patient-level mean tacrolimus trough concentration during the 90 days preceding the index date, summarized as the median (IQR) for each study group; MMF, mycophenolate mofetil; SRL, sirolimus; DM, diabetes mellitus; HTN, hypertension; SD, standard deviation; IQR, interquartile range; ns, not significant.

Cox regression analysis identified older age at transplantation as the strongest independent predictor of PTM (HR 1.08 per year, 95% CI 1.05–1.11, p < 0.001) and all-cause mortality (HR 1.07 per year, 95% CI 1.04–1.10, p < 0.001) in the overall cohort (**Table 4**). Male sex and diabetes were associated with higher mortality in univariable analysis but did not remain significant after adjustment. Higher long-term tacrolimus trough levels were independently associated with mortality (HR 1.24, p = 0.028), whereas long-term tacrolimus trough levels were not independently associated with PTM development after multivariable adjustment.

**Table 4:** Risk factors for cancer development and mortality among kidney transplant recipients (N = 625).

Variable	Cancer development – Univariable HR (95% CI)	p	Cancer development – Multivariable HR (95% CI)	p	Mortality – Univariable HR (95% CI)	p	Mortality – Multivariable HR (95% CI)	p
Sex (Male *vs* Female)	1.15 (0.69–1.90)	0.592	1.21 (0.67–2.19)	0.536	1.71 (1.06–2.76)	0.027*	1.44 (0.78–2.66)	0.242
Age_KT	1.08 (1.05–1.10)	<0.001**	1.08 (1.05–1.11)	<0.001**	1.07 (1.05–1.10)	<0.001**	1.07 (1.04–1.10)	<0.001**
Age_C	0.98 (0.96–1.00)	0.1	–	–	1.03 (0.99–1.08)	0.167	–	–
Cancer (Yes *vs* No)	–	–	–	–	1.80 (1.02–3.16)	0.042*	1.41 (0.69–2.88)	0.346
LFK1	0.96 (0.86–1.08)	0.489	–	–	1.06 (1.01–1.12)	0.019*	1.24 (1.02–1.50)	0.028*
LFK2	0.95 (0.84–1.07)	0.426	–	–	1.05 (0.99–1.11)	0.134	–	–
LFK3	1.01 (0.91–1.11)	0.905	–	–	0.94 (0.81–1.11)	0.475	–	–
LFK4	0.99 (0.96–1.03)	0.643	–	–	1.03 (1.01–1.05)	0.010**	0.99 (0.94–1.03)	0.621
SFK1	0.85 (0.73–0.99)	0.040*	1.41 (0.97–2.05)	0.069	1.13 (1.04–1.24)	0.005**	0.84 (0.53–1.34)	0.463
SFK2	0.82 (0.70–0.96)	0.015*	0.68 (0.42–1.10)	0.118	1.10 (0.98–1.24)	0.102	–	–
SFK3	0.80 (0.67–0.96)	0.014*	0.83 (0.60–1.14)	0.245	0.84 (0.71–0.99)	0.038*	0.78 (0.53–1.13)	0.188
SFK4	0.95 (0.86–1.05)	0.299	–	–	1.08 (1.05–1.10)	<0.001**	1.12 (0.96–1.31)	0.161
DM (overall)	1.04 (0.63–1.71)	0.887	–	–	1.84 (1.16–2.92)	0.009**	1.17 (0.64–2.14)	0.604
HBV (overall)	–	–	–	–	–	–	–	–
HCV (overall)	1.30 (0.64–2.64)	0.475	–	–	1.15 (0.57–2.31)	0.692	–	–
HTN (overall)	1.18 (0.66–2.12)	0.572	–	–	1.02 (0.61–1.70)	0.939	–	–

Age_KT, age at kidney transplantation; age_C, age at cancer diagnosis; LFK, long-term tacrolimus trough concentration measured from kidney transplantation to the index date (PTM diagnosis for recipients with PTM or last follow-up for recipients without PTM); SFK, short-term tacrolimus trough concentration measured during the 90 days preceding the index date; LFK1–4 and SFK1–4 denote the mean, median, minimum, and maximum trough concentrations, respectively; DM, diabetes mellitus; HBV, hepatitis B virus; HCV, hepatitis C virus; HTN, hypertension; HR, hazard ratio; CI, confidence interval.

Statistical method: Cox proportional hazards regression analysis. *p < 0.05, **p < 0.01.

In the PTM subgroup (n = 62), older age predicted both cancer progression or recurrence (HR 1.03, 95% CI 1.00–1.05, p = 0.044) and mortality (HR 1.07, 95% CI 1.02–1.13, p = 0.008). Higher long-term tacrolimus trough levels remained independently associated with mortality (HR 1.37, 95% CI 1.03–1.82, p = 0.031). No other demographic or clinical factors reached statistical significance in multivariable analysis (**Table 5**).

**Table 5:** Risk factors for cancer development and mortality among kidney transplant recipients with PTM (N = 62).

Variable	Cancer development – Univariable HR (95% CI)	p	Cancer development – Multivariable HR (95% CI)	p	Mortality – Univariable HR (95% CI)	p	Mortality – Multivariable HR (95% CI)	p
Sex (Male *vs* Female)	1.16 (0.70–1.93)	0.559	0.88 (0.50–1.53)	0.642	1.58 (0.54–4.63)	0.407	1.03 (0.34–3.17)	0.954
Age_KT	1.02 (1.00–1.05)	0.07	1.03 (1.00–1.05)	0.044*	1.07 (1.02–1.12)	0.006**	1.07 (1.02–1.13)	0.008**
Age_C	0.98 (0.96–1.00)	0.1	–	–	1.03 (0.99–1.08)	0.167	–	–
LFK1	1.28 (1.12–1.46)	<0.001**	0.70 (0.19–2.58)	0.587	1.33 (1.01–1.75)	0.040*	1.37 (1.03–1.83)	0.031*
LFK2	1.31 (1.13–1.50)	<0.001**	1.67 (0.48–5.88)	0.422	1.30 (0.98–1.74)	0.072	–	–
LFK3	1.46 (1.22–1.74)	<0.001**	1.19 (0.84–1.67)	0.327	1.41 (0.90–2.22)	0.137	–	–
LFK4	1.05 (1.01–1.09)	0.007**	1.03 (0.93–1.13)	0.575	1.05 (0.98–1.13)	0.127	–	–
SFK1	1.10 (0.97–1.26)	0.146	–	–	1.19 (0.93–1.51)	0.164	–	–
SFK2	1.14 (0.97–1.33)	0.102	–	–	1.27 (0.94–1.71)	0.113	–	–
SFK3	1.24 (0.99–1.55)	0.057	–	–	1.50 (0.96–2.37)	0.077	–	–
SFK4	1.04 (0.97–1.11)	0.319	–	–	1.06 (0.94–1.19)	0.335	–	–
DM (overall)	0.63 (0.38–1.05)	0.079	–	–	0.40 (0.14–1.18)	0.098	–	–
HBV (overall)	–	–	–	–	–	–	–	–
HCV (overall)	1.06 (0.52–2.16)	0.872	–	–	1.42 (0.40–5.03)	0.591	–	–
HTN (overall)	0.84 (0.47–1.52)	0.561	–	–	0.37 (0.13–1.06)	0.064	–	–

Age_KT, age at kidney transplantation; age_C, age at cancer diagnosis; LFK, long-term tacrolimus trough concentration measured from kidney transplantation to the index date (PTM diagnosis); SFK, short-term tacrolimus trough concentration measured during the 90 days preceding the index date; LFK1–4 and SFK1–4 denote the mean, median, minimum, and maximum trough concentrations, respectively; DM, diabetes mellitus; HBV, hepatitis B virus; HCV, hepatitis C virus; HTN, hypertension; HR, hazard ratio; CI, confidence interval.

Statistical method: Cox proportional hazards regression analysis. *p < 0.05, **p < 0.01.

### Survival outcomes

Among PTM patients, 46.3% died during follow-up. Median survival after cancer diagnosis was 16.5 months (IQR 7.0–47.8), and median survival from transplantation to death was 10.5 years (IQR 6.0–14.0). Male PTM patients had higher mortality than females (54.6% *vs*. 36.8%). Higher long-term tacrolimus trough levels remained independently associated with mortality in both the overall and PTM cohorts (**Tables 4**, **5**).

## Discussion

### Global and regional perspectives on PTM incidence

Post-transplant malignancy remains one of the most serious late complications in kidney transplant recipients, ranking second only to cardiovascular disease and infection as a cause of death (4–7). Reported incidences vary widely between regions. Western cohorts typically report rates between 10% and 20%, with non melanoma skin cancers and lymphomas predominating (5, 12–17). In contrast, Asian studies consistently show lower incidences, ranging from 2% to 7.7% (3, 14–17, 41). National data from Taiwan between 1997 and 2008 estimated incidences of 6.8% to 8.4% (10, 24–26). The present 20-year single center cohort revealed an incidence of 11.7%, aligning more closely with Western reports (2, 5). This likely reflects extended follow up, comprehensive coverage under Taiwan’s National Health Insurance, and improved diagnostic sensitivity. Recent regional analyses suggest a gradual convergence of PTM incidence toward Western levels as improved survival prolongs cumulative immunosuppressive exposure in Asian transplant populations (5, 6). From an immunologic standpoint, this pattern is consistent with sustained impairment of cancer immunoediting and immune metabolic exhaustion under chronic immunosuppression (20, 28, 29).

### Cancer spectrum and environmental determinants in Taiwan

The cancer spectrum in our cohort highlights the region specific interplay between environmental carcinogens and immunologic suppression. In contrast to Western populations, where non melanoma skin cancer dominates (5, 12–17), urothelial and hepatic cancers were the most frequent PTMs in Taiwanese recipients, consistent with prior Asian and Taiwanese series (3, 10, 14–17, 24–27). Aristolochic acid exposure produces characteristic DNA adducts and TP53 mutational signatures that drive upper tract urothelial carcinoma (21, 22), while arsenic exposure has been linked to urothelial and cutaneous malignancies (23). Genetic predispositions, including variants in detoxification pathways, may modulate susceptibility (7–9, 24, 25). Regional cofactors such as chronic HBV and HCV further compound risk by sustaining hepatic inflammation and oncogenesis, while toxin related DNA damage synergizes with impaired immune clearance to accelerate tumorigenesis (2, 7, 11, 20).

The intermediate non melanoma skin cancer rate in our series likely reflects expanded dermatologic surveillance under National Health Insurance rather than a true increase in disease burden. From an immunologic perspective, chronic toxin induced DNA damage may synergize with reduced tumor immune surveillance to accelerate oncogenesis (28, 30).

### Sex-specific disparities and immunologic basis

Sex-specific differences were evident. Female recipients more often developed urothelial cancers, whereas male recipients more frequently developed hepatic, renal, and cutaneous malignancies, mirroring other Asian reports (3, 14–17). Differential exposures such as tobacco and occupational carcinogens, higher background rates of chronic viral hepatitis in men, and hormonal or immunogenetic modifiers likely contribute (2, 7, 11, 24). Sex hormones shape antitumor immunity, with estrogen enhancing CD8^+^ effector programs and androgens favoring regulatory phenotypes and T cell exhaustion, offering a mechanistic basis for the observed disparities (20, 25, 26). These data support sex specific surveillance with heightened attention to urothelial malignancies in women and hepatocellular carcinoma in men.

### Temporal shifts and evolving risk landscape

A marked decline in bladder cancer incidence was observed after 2012, consistent with earlier Taiwanese observations that identified bladder cancer as a dominant PTM (10, 27). Multiple factors likely contributed, including gradual reduction in calcineurin inhibitor exposure and broader adoption of mTOR inhibitors with antiproliferative effects, as well as structured urologic surveillance (18, 19, 35). Concurrent public health actions reduced exposure to urothelial carcinogens through prohibition of aristolochic acid containing herbal products and remediation of arsenic contaminated groundwater (21–23). Nevertheless, recipients transplanted after 2012 had a shorter potential follow-up period than those transplanted before 2012, which may have resulted in underascertainment of late-onset malignancies and influenced the observed proportions across the two eras. Therefore, these temporal trends should be interpreted with appropriate caution. Similar patterns have been described regionally following aristolochic acid control and greater use of mTOR based strategies, underscoring how pharmacologic refinement and environmental regulation can reshape transplant oncology outcomes (5, 6). By contrast, incidences of liver, kidney, and skin cancers remained stable, and colorectal cancer appeared in the contemporary era in parallel with aging recipients and wider colonoscopy screening (27). These shifts illustrate how environmental intervention and immune modulation can reshape cancer epidemiology in transplant populations (25, 35).

### Age, immunosenescence, and cumulative immunosuppression

Older age at transplantation was the strongest predictor of PTM and mortality, in line with reports from the United States, Korea, and Europe (9, 15, 17, 30, 37). Immunosenescence reduces cytotoxic T cell function, narrows clonal diversity, and increases regulatory T cell dominance, thereby weakening tumor immune surveillance (29, 31). Age related immune remodeling encompasses metabolic exhaustion and diminished interferon signaling, likely compounding oncogenic risk in older recipients under chronic immunosuppression (20). Cumulative immunosuppressive exposure was also decisive: most PTM patients received three or more agents, and risk rose with five or more, supporting evidence that overall immunosuppressive intensity rather than any single agent drives oncogenesis (7, 10, 36–38). Although hydrocortisone and sirolimus use was more common in PTM patients, the latter likely reflects indication bias because sirolimus is frequently introduced after cancer diagnosis for antiproliferative benefit (18, 19, 35).

### Tacrolimus exposure and immune surveillance dysfunction

The oncogenic role of tacrolimus remains complex. Meta analyses and experimental studies associate calcineurin inhibition with increased malignancy risk via TGF beta signaling and mTOR pathway activation (32–34). Other analyses emphasize cumulative exposure over specific drug choice (10, 36). Mechanistic data indicate that chronic calcineurin blockade drives metabolic reprogramming of effector T cells toward exhaustion and impairs dendritic cell antigen presentation, collectively undermining immune surveillance (20).

In our cohort, PTM recipients had lower long-term (LFK) and short-term (SFK) mean tacrolimus trough concentrations than non-PTM recipients. Importantly, these measures were calculated up to the predefined index date—PTM diagnosis for recipients with malignancy and the last follow-up date for recipients without malignancy—and therefore should not be interpreted as evidence that lower tacrolimus exposure increases PTM risk. Rather, they reflect differences in longitudinal tacrolimus exposure before the respective index dates and may also have been influenced by differences in follow-up duration, immunosuppressive management, and patient characteristics.

The paired longitudinal analysis addressed a distinct clinical question. Among 44 PTM recipients with paired data, tacrolimus trough levels were significantly lower during the year after PTM diagnosis than during the year before diagnosis, consistent with routine reduction of immunosuppression following cancer recognition. These findings indicate that pre-diagnosis tacrolimus exposure and post-diagnosis dose reduction represent separate clinical processes and should be interpreted independently. Furthermore, higher long-term tacrolimus trough levels were independently associated with all-cause mortality in multivariable models, supporting a dose- and context-dependent association between tacrolimus exposure and adverse outcomes and highlighting the importance of individualized therapeutic drug monitoring (25, 32–34).

Although our findings do not establish a therapeutic tacrolimus threshold, they suggest that longitudinal monitoring of tacrolimus trough levels may provide more clinically meaningful information than isolated measurements when evaluating long-term outcomes after kidney transplantation. Individualized therapeutic drug monitoring should therefore balance adequate graft immunosuppression against long-term malignancy risk while maintaining optimal graft function. These observations should be considered hypothesis-generating and warrant prospective validation using standardized longitudinal tacrolimus exposure metrics.

### Prognostic impact and immune exhaustion

Mortality was substantially higher in PTM patients than in non-PTM recipients, consistent with registry data showing malignancy accounts for a large proportion of late post-transplant deaths (37, 38). Post-diagnosis survival was short, indicating aggressive disease behavior under chronic immunosuppression, and male sex was associated with worse outcomes, echoing prior reports (2, 24).

Poor outcomes are linked to persistent exhaustion signatures in tumor-reactive T cells, including PD-1, TIM-3, and LAG-3 upregulation (20, 39). Nonetheless, median overall survival from transplantation of 10.5 years indicates that durable benefit remains attainable with vigilant detection and management (37). Higher long-term tacrolimus trough levels remained independently associated with mortality, underscoring the importance of individualized therapeutic drug monitoring while balancing adequate graft immunosuppression against long-term malignancy risk (25, 35, 36). Balancing graft tolerance against antitumor immunity remains challenging. Immune checkpoint blockade may restore cytotoxicity but carries a substantial risk of allograft rejection in transplant recipients (39–41).

### Limitations

This study has several limitations. The retrospective single center design introduces potential selection and information biases, and the extended study period encompassed major changes in immunosuppressive and oncologic practice. Longitudinal tacrolimus trough data were available only for a subset of recipients, and trough concentrations may not fully reflect cumulative immunosuppressive exposure. Environmental and viral cofactors such as Epstein–Barr virus, human papillomavirus, and hepatitis viruses were not systematically evaluated. Mortality ascertainment in non PTM patients was incomplete, and findings may not be generalizable beyond the Taiwanese population. Future multicenter collaborations incorporating longitudinal immune phenotyping and molecular carcinogen profiling are warranted to validate these findings and further clarify the mechanisms underlying immune exhaustion and cancer risk after transplantation.

### Clinical implications and future directions

The distinct cancer spectrum observed in this Taiwanese cohort underscores the importance of region- and sex-specific surveillance strategies, with intensified screening for urothelial malignancies in women and hepatocellular carcinoma in men. The decline in bladder cancer after 2012 illustrates how immunosuppressive optimization and environmental regulation can effectively reduce cancer burden in transplant recipients. The associations between cumulative immunosuppressive burden and PTM development, together with the independent association between higher long-term tacrolimus trough levels and mortality, highlight the need for individualized, adaptive immunosuppressive monitoring. Emerging evidence supports integrating immune functional assays, cytokine profiling, and viral load monitoring into routine post-transplant care to improve cancer risk stratification (42). In the long term, personalized immunosuppressive protocols guided by immune senescence and checkpoint expression profiles may allow precision immunomodulation that balances graft tolerance with effective tumor surveillance.

## Conclusions

This large-scale longitudinal study provides one of the most comprehensive evaluations of post-transplant malignancy (PTM) patterns in an Asian kidney transplant population.

In our two-decade Taiwanese cohort, the PTM incidence reached 11.7%, exceeding previous Asian estimates and characterized by a distinct spectrum dominated by urothelial and hepatic cancers. A significant decline in bladder cancer incidence after 2012 may reflects evolving immunosuppressive strategies, structured surveillance, and environmental interventions.

Older age at transplantation and cumulative immunosuppressive burden were associated with PTM development, whereas higher long-term tacrolimus trough levels were independently associated with mortality, underscoring the importance of individualized therapeutic drug monitoring.

Although PTM substantially increased mortality risk, long-term post-transplant survival remained achievable. These findings emphasize the need for region-specific surveillance and individualized immunosuppressive optimization to enhance long-term outcomes in kidney-transplant recipients and inform global cancer-prevention strategies in transplant populations.

## Data Availability

The original contributions presented in the study are included in the article/**Supplementary Material**. Further inquiries can be directed to the corresponding authors.
