# Supplementary material for: Post-transplant malignancies in Taiwanese kidney transplant recipients: incidence, temporal trends, and immunosuppressive risk factors in a two-decade single-center cohort
Source: Front Immunol. 2026 Aug 17;17:1738516. doi: 10.3389/fimmu.2026.1738516 (PMC13526915; doi:10.3389/fimmu.2026.1738516)
Supplement: Supplementary file 1 [file DataSheet1.pdf]

Supplementary Table S1. Time to onset of post-transplant malignancies by cancer type

Cancer type	Median (years)	Q1 (years)	Q3 (years)	N
Urothelial carcinoma (all)	8.03	4.66	12.16	61
Bladder	8.99	4.51	12.25	32
Ureter	8.50	6.49	14.33	16
Renal pelvis	7.71	4.65	8.20	13
Upper tract UC (UTUC)	7.91	5.84	10.83	29
Liver	7.57	3.04	11.99	25
Kidney	7.61	3.50	10.54	17
Skin (NMSC)	7.14	3.56	9.86	17
Colorectal	9.58	4.34	11.93	15
Lung	7.84	3.66	10.51	11
Breast	5.64	2.88	9.56	8
Prostate	9.06	5.44	9.56	8

Data are presented as median years from kidney transplantation to cancer diagnosis, with first (Q1) and third (Q3) quartiles. UC: urothelial carcinoma; UTUC: upper tract urothelial carcinoma; NMSC: non-melanoma skin cancer.

Supplementary Table S2. Temporal Distribution of Post-Transplant Malignancies Before and After June 2012 (N = 203)

Variable	After June 2012 (n=143)		Before June 2012 (n=60)		*p* value
	Count	%	Count	%	
sex					0.068†
Female	61	43%	34	57%	
Male	82	57%	26	43%	
Age at KT, mean ± SD	50.82 ±12.67		51.48 ±9.12		0.677
Age at cancer, median (IQR)	62.00 (53.0-66.0)		56.50 (54.0-66.5)		0.000***
Deceased	57	40%	20	33%	0.382
Comorbidity					
DM	70	49%	22	37%	0.109
HBV	33	23%	10	17%	0.308
HCV	28	20%	6	10%	0.095†
HTN	106	74%	46	77%	0.703
CAD	51	36%	16	27%	0.214
COPD	17	12%	4	7%	0.265
CVA	17	12%	5	8%	0.457
HLD	95	66%	38	63%	0.672
IgAN	5	3%	1	2%	0.672
PKD	8	6%	2	3%	0.726
SLE	8	6%	1	2%	0.286
Cancer type					
Bladder	16	11%	16	27%	0.010
Bone marrow	1	1%	0	0%	1.000
Border of tongue	1	1%	0	0%	1.000
Brain	2	1%	2	3%	0.583
Breast	6	4%	2	3%	1.000
Cervix	2	1%	1	2%	1.000
Colon	9	6%	0	0%	0.060
Connective tissue	1	1%	0	0%	1.000
Endometrium	1	1%	1	2%	0.505
Extrahepatic bile duct	1	1%	0	0%	1.000
Gallbladder	1	1%	0	0%	1.000
Intrahepatic bile duct	1	1%	0	0%	1.000
Kidney	10	7%	7	12%	0.278
Liver	17	12%	8	13%	0.816
Lung	10	7%	1	2%	0.180
Lymph nodes	2	1%	0	0%	1.000
Malignant neoplasm of cheek mucosa	1	1%	0	0%	1.000
Malignant neoplasm of mouth	1	1%	0	0%	1.000
Nasopharynx	0	0%	1	2%	0.296
Oropharynx	1	1%	0	0%	1.000
Pancreas	4	3%	0	0%	0.322
Parotid gland	1	1%	0	0%	1.000
Penis	1	1%	0	0%	1.000
Prostate gland	5	3%	3	5%	0.696
Rectal	3	2%	3	5%	0.363
Renal pelvis	8	6%	5	8%	0.532
Skin	11	8%	6	10%	0.587
Stomach	1	1%	1	2%	0.505
Thoracic esophagus	1	1%	0	0%	1.000
Thyroid gland	5	3%	0	0%	0.324
Unknown primary site	3	2%	0	0%	0.557
Ureter	13	9%	3	5%	0.404
Vulva	3	2%	0	0%	0.557
UTUC	21	15%	8	13%	0.557

Abbreviations: KT, kidney transplantation; IQR, interquartile range; DM, diabetes mellitus; HBV, hepatitis B virus; HCV, hepatitis C virus; HTN, hypertension; CAD, coronary artery disease; COPD, chronic obstructive pulmonary disease; CVA, cerebrovascular accident; HLD, hyperlipidemia; IgAN, IgA nephropathy; PKD, polycystic kidney disease; SLE, systemic lupus erythematosus; UTUC, upper tract urothelial carcinoma.

Statistical method: Data are shown as n (%) or mean ± SD / median (IQR). Comparisons were performed using chi-square test (or Fisher's exact test if expected count < 5) for categorical variables, and Welch's t-test for continuous variables. *p < 0.05, **p < 0.01. †

trend-level significance ($0.05 \leq p < 0.10$).

Supplementary Table S3. Ever-use of individual immunosuppressive agents in kidney transplant recipients with and without PTM

Variable	Total (N = 1,476)	Total %	No PTM (N = 1,281)	No PTM %	PTM (N = 195)	PTM %	p value
Methylprednisolone	607	41.1	530	41.4	77	39.5	0.618
Hydrocortisone	657	44.5	524	40.9	133	68.2	<0.001 **
Prednisolone	677	45.9	595	46.5	82	42.1	0.251
Tacrolimus	1,179	79.9	1,023	79.9	156	80.0	0.964
Mycophenolate	1,317	89.2	1,138	88.8	179	91.8	0.215
Sirolimus	422	28.6	331	25.8	91	46.7	<0.001 **
Everolimus	1	0.1	0	0.0	1	0.5	0.132

Chi-square test or Fisher's exact test, as appropriate. *P* < 0.05; **P** < 0.01.
