# Supplementary material for: Post-transplant malignancies in Taiwanese kidney transplant recipients: incidence, temporal trends, and immunosuppressive risk factors in a two-decade single-center cohort
Source: Front Immunol. 2026 Aug 17;17:1738516. doi: 10.3389/fimmu.2026.1738516 (PMC13526915; doi:10.3389/fimmu.2026.1738516)
Supplement: Supplementary file 2 [file DataSheet2.pdf]

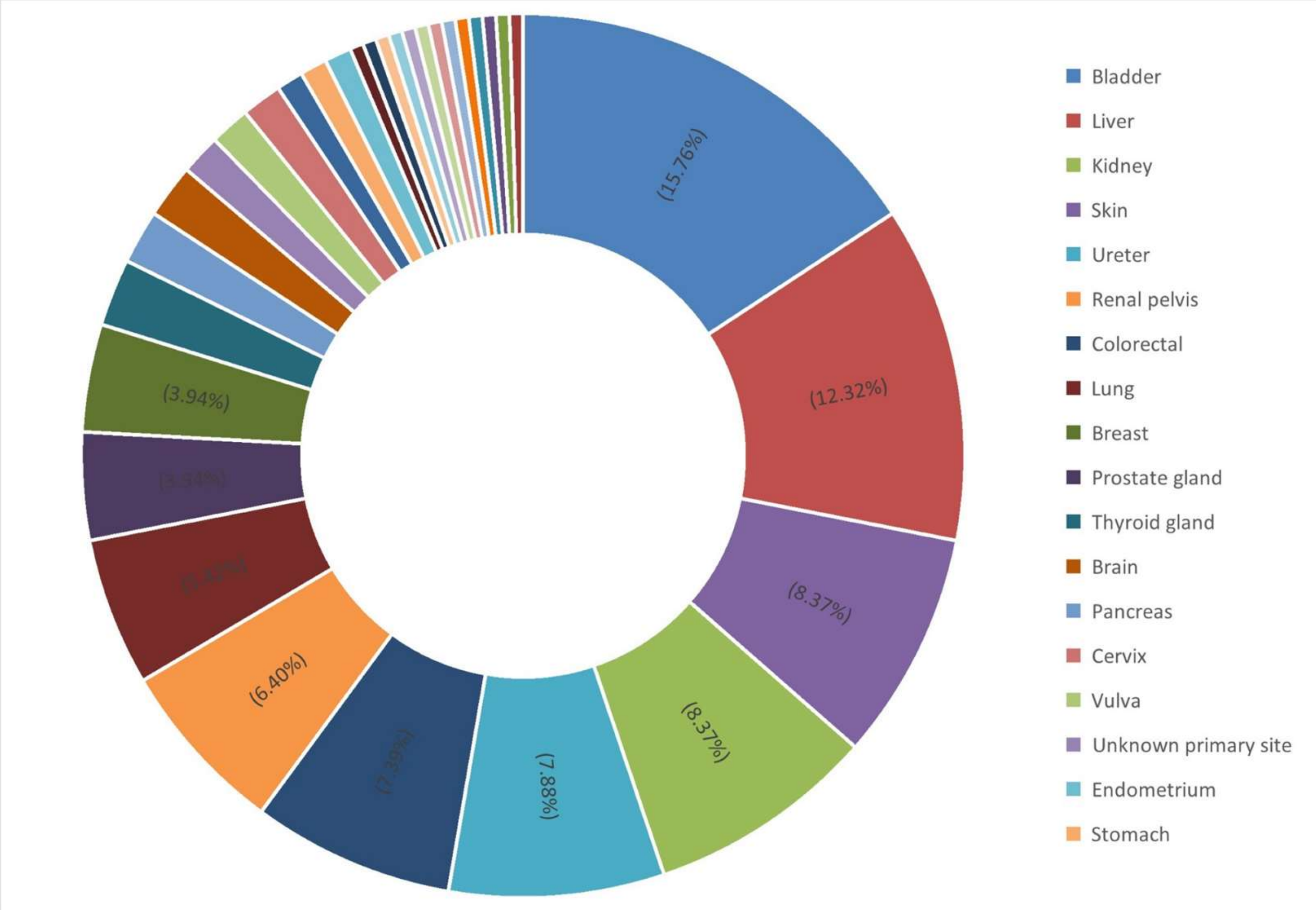

Supplementary Figure S1. Proportional distribution of the ten most common PTM subtypes in the overall cohort.

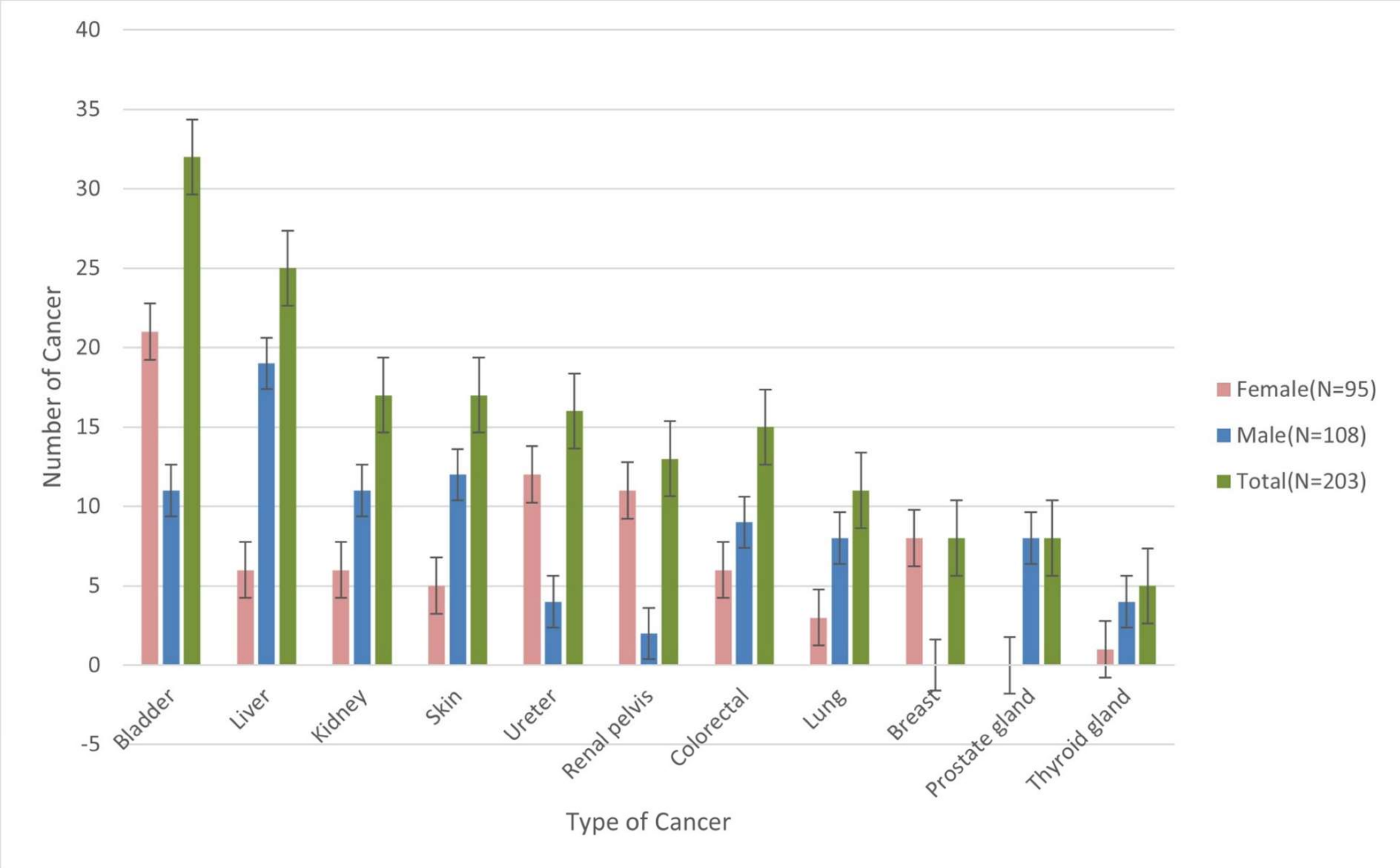

Supplementary Figure S2. Sex-specific bar charts of PTM distribution (male vs. female).

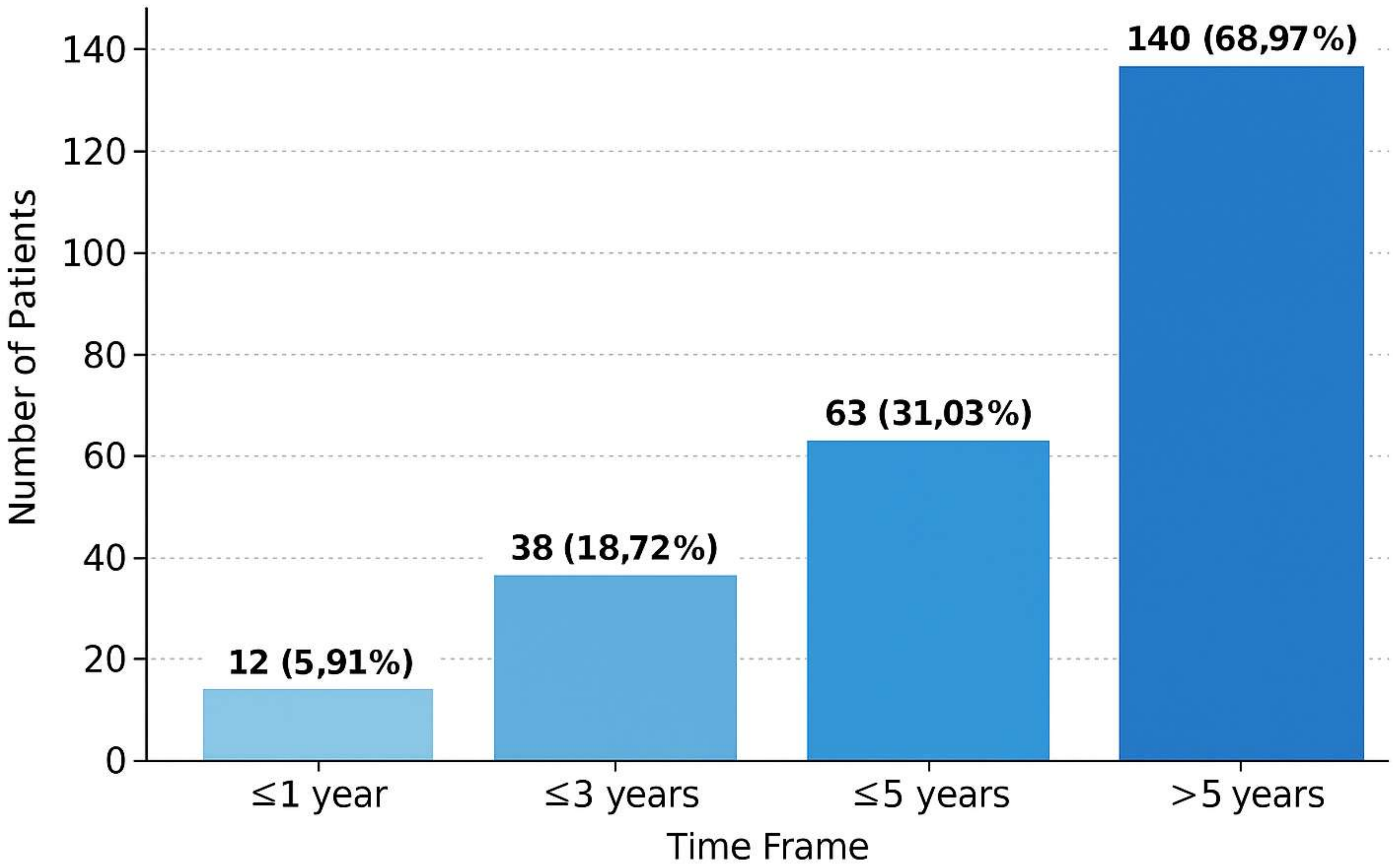

Supplementary Figure S3. Distribution of time from kidney transplantation to the diagnosis of post-transplant malignancy (PTM).

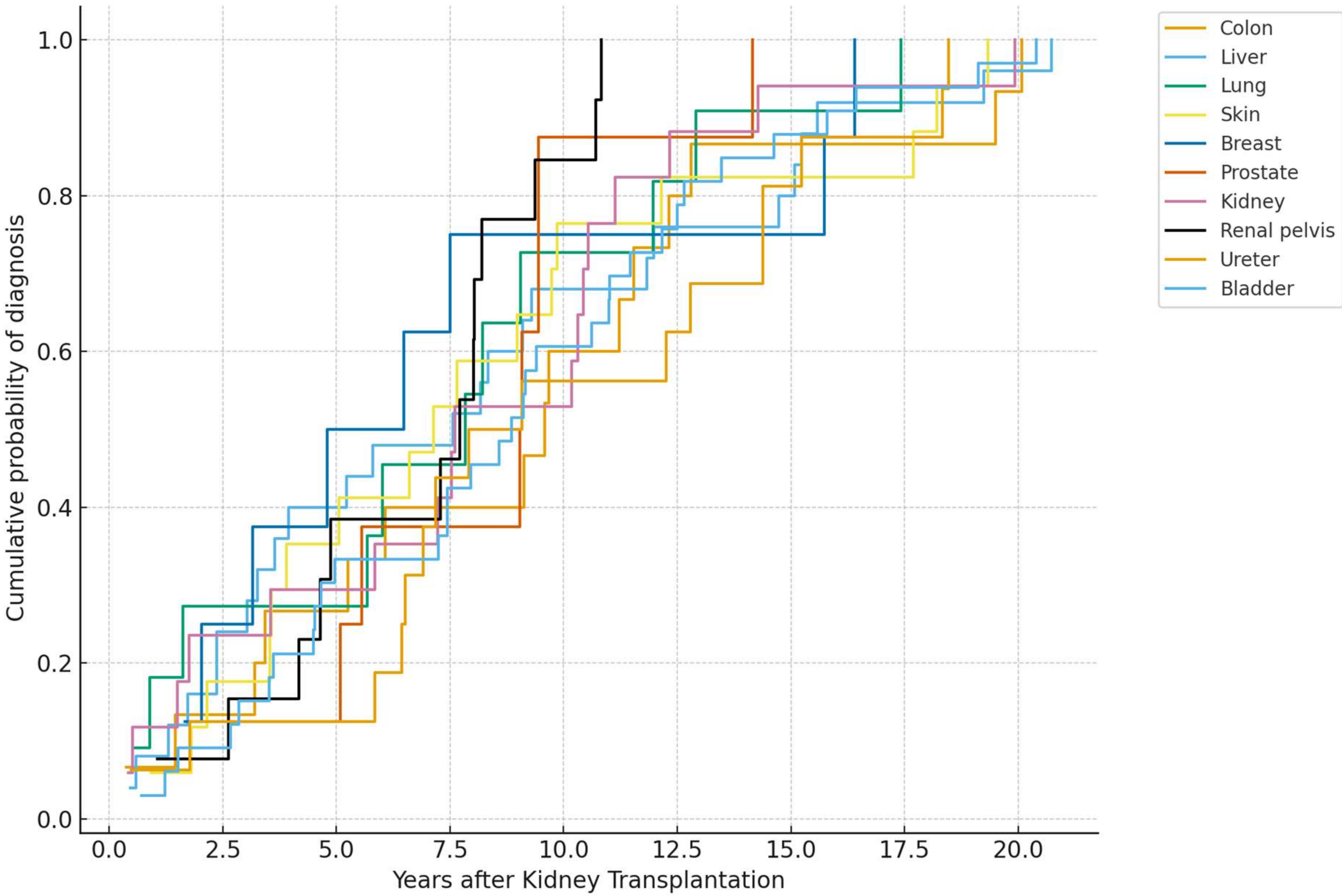

Supplementary Figure S4. Cumulative incidence of post-transplant malignancy stratified by cancer type.
